# Supplementary material for: “We only trust each other”: A qualitative study exploring the overdose risk environment among persons who inject drugs living with HIV in Nairobi, Kenya
Source: PLOS Glob Public Health. 2024 Jul 2;4(7):e0003435. doi: 10.1371/journal.pgph.0003435 (PMC11218959; doi:10.1371/journal.pgph.0003435)
Supplement: Attachment — Submitted filename: ResponsetoReviewers_Maurano.docx [file pgph.0003435.s002.docx]

**Response to Reviewers**

We appreciate the thoughtful comments of the reviewers, and we feel that their suggestions have substantially strengthened and clarified our manuscript. We welcome the opportunity to specifically address all of their comments. Below we address the editor’s and reviewers’ comments point-by-point and reference where the revisions can be found within the manuscript.

**EDITOR’S COMMENTS**

1. The first author and co-senior authors are all affiliated with US institutions. As this is a study in Kenya, senior authorship should include a Kenyan author or an author affiliated with one of the Kenyan institutions.

Thank you for leading the way for global health equity as we asked a co-author, who was instrumental in our work to serve as a senior author. Please see the author on our Title Page. (Page 1, Line 17)

2. Which author is affiliated with [i) Institute of Public Health Genetics, School of Public Health, University of Washington, UW Box 33 # 351619, Seattle, WA, 98195, USA] as this is not listed next to any of the authors names?

We apologize for this oversight and thank the editor for catching this typo. One of our co-authors was partially funded by the Institute of Public Health Genetics; however, they were not funded during the project period and we have removed this affiliation. (Page 1, Line 31-32)

3. Please see the comments from reviewers below, please make the changes as listed for acceptance. Please note from reviewer 2 comments, from my perspective as editor, the inclusion of the interview script is not required for acceptance but a summary figure with the main themes as sub-themes would be helpful.

We agree with the editor that providing a summary table of the main themes and brief definitions of each theme increases visibility and transparency of our findings to the PLOS readership. We opted not to include the interview guide, but would be happy to share this material upon request. Please note the inclusion of an additional table prompted us to re-number our tables. Please see Table 1, located on page 8, lines 221-237.

**REVIEWER 1 COMMENTS**

Review summary: Overall, the paper reads well and is a good addition to the evidence available in Kenya and the region regarding overdose occurrence, prevention and response.

1. Consider re-structuring the sentence line 87-89 on opioid overdose being the leading cause of mortality among PWID in Kenya. The referenced article only indicates the percentage of opioid overdose reported (40% in Kenya), that it is a leading cause (from a meta-analysis).

We appreciate the reviewer raising this point of clarification and allowing us to revise the sentence. Reference 4 supports the claims that 40% of PWID report personal overdoses according to a national poll conducted by Kenya’s Ministry of Health (MOH) Key Populations Programme and the National AIDS and STI Control Programme (NASCOP). The MOH 2021 report indicates that overdose is a leading cause of morbidity and mortality among PWID in the region. Additionally, we separated this from our claim that living with HIV increases the risk of experiencing both fatal and nonfatal overdose, and provide the appropriate reference by Green et al. You can find this revision on Page 3 (Line 91-93), which reads:

*“Overdose is a leading cause of mortality among PWID in Kenya, with approximately 40% of PWID reporting experiencing a personal overdose [4], which may be higher for PWID living with HIV [5].”*

2. Line 153 make a correction on the reimbursement amount, as the Ksh value has a dollar sign on it as well.

We have addressed the reviewer’s comment on Page 6, Line 167, which now reads:

*“Participants provided written informed consent in Swahili or English and were reimbursed 400 Ksh ($4 USD) for their time and transportation.”*

3. Line 269-273- the quote is not indicative of overdose, but rather the effect of an unknown substance

We agree that this was not an exemplar quote that showcased the main point of our theme of “overdose precursors”. We do believe that PWID are at risk of experiencing an overdose when they are dependent on someone else purchasing their drugs, which we clarified with in the body of text. However, we chose to use a more robust quote from a participant with multiple overdose experiences. Our edits can be located on Page 12, Line 302-312.

*“Jimiyu (M, 41 yrs) has used heroin since the age of 17 and described multiple overdose experiences. His first overdose occurred within the first year of injecting drugs, where he injected too much heroin at once, and another time he describes polysubstance use of drinking alcohol and injecting heroin. Jimiyu describes his most recent overdose, which he attributes to his high opioid tolerance and forgetting to track his daily heroin intake:*

*‘I felt that the steam had gone low and I even used to forget, I had forgotten, that I had taken it earlier and I go back to get it again. My brain is becoming slower and cannot cooperate as much as it used to.’ - Jimiyu (M, 41 yrs)*

*This highlights overdose risk may be different for PWID with varying years of experience injecting drugs.”*

4. Line 415- were there examples of stimulants injected that were provided? There is need to mention these

We completely agree with the notion that injecting opioids, compared with stimulants, has very different drug-related consequences and prevention strategies. We were able to characterize our participants’’ drug use in Table 2, which highlights the majority of our participants reported heroin use (79%) and a small proportion indicated cocaine use (21%). This was ascertained by asking participants to list all of the substances they consumed in the previous year, that were not prescribed by a medical professional. While the most of the overdose symptoms described align with opioid overdose symptoms, we acknowledge that this is a limitation of our study (Page 24, Lines 646-649). We also acknowledge that 79% of our participants reported using multiple substances together or consecutively (i.e., polysubstance use), which suggests that it may be difficult to distinguish overdose type.

5. 427 grammatical correction.

We have edited this statement (Page 18, Lines 489-493) to read:

*“Naloxone is available in densely populated injection settings like dens that contain 20 to 300 PWID and dealers, through peer educators, pharmacies and clinics known to treated PWID patients. However, these points of access may be of limited utility as harm reduction facilities, peer educators, clinics, and pharmacies have limited service hours in the evenings and on weekends.”*

6. Overall, were there specific shared experiences of heroin methadone polyuse and overdose?

We thank our review as this is a great point of distinction. We suspect that heroin and methadone use was more prevalent in the past, but most participants knew that they were at increased risk for experiencing an overdose if they combined heroin and methadone. A couple of years prior to our interviews, harm reduction facilities and methadone facilities modified their protocols to include education and counselling on overdose risks attributed to combining heroin and methadone use, and methadone clinics regularly test methadone patients for heroin use. If patients receive a positive drug test, they are counselled and educated on the risks of overdose. We felt this comment provided an opportunity to showcase the benefits of educating PWID communities of overdose risks and modified the following sentences (Page 15, 441-442):

*“Similarly, nine participants credited peer educators, peers, and OAT clinical staff who educated them on the increased overdose risk when combining heroin and methadone. Findings point to the recently implemented educational campaigns that highlight overdose risks of taking both methadone and heroin are reaching PWID communities.”*

**REVIEWER 2 COMMENTS**

Review Summary: This paper aims to explore the characteristics of overdose risk environments for people who inject drugs and are living with HIV in Kenya, with the goal of identifying recommendations to improve existing policies and services for reducing overdose risks. This qualitative study is well-conducted. Below, authors can find my comments aimed at improving the paper:

1. Pg 3: keywords - As PWID is used as a keyword for indexing, it might be better to have it listed separately, outside of brackets.

Thank you for this suggestion, we have made edits to our list of keywords to improve visibility (Page 2, Lines 51-52)

2. Pg 4: In the introduction's first paragraph, you provide information about different drugs such as opioids or heroin (referred to as white crest and brown sugar). However, switching between different drugs may make it somewhat challenging for the reader to follow. Authors may consider focusing on one type of drug to effectively highlight the problem.

As a team, we reviewed the transcripts several times to determine if we could identify all substances consumed prior to each overdose. The personal overdoses were mostly heroin overdoses; however, we were not able to fully ascertain the cause of observed overdoses, which we mention in the limitations section (Page24, Lines 646-649). We do know that heroin is dominate within the region and most of the described overdose symptoms align with opioid overdoses. In the introduction, we thought it would be beneficial to provide historical context to the shifting drug market. While this may be limitation, my co-authors and I feel that the contributions of our data, can assist with intervention development and highlights a critical need for additional research on substances taken prior to an overdose.

3. Pg 5: You've hinted at reasons for choosing the risk environment framework, but it could be clearer for the reader why this framework was chosen for your study. Providing more explicit rationale for selecting this framework would enhance the clarity and understanding of your research approach.

We would like to thank the review for allowing us to expand our rationale for applying the overdose risk environment framework, which has largely been applied to high- and middle-income countries and has yet to be applied within Kenya. We have made an adjustment to our paragraph introducing the risk environment, with the added sentence underlined (Page 4, Lines 115-120):

*“Using a risk environment framework provides a conceptual overview of how an individual can influence and be influenced by their surrounding physical, social, economic, and political environments, which sustain drug-related morbidity and mortality [12,13]. Specifically, the risk environment framework proposed by Rhodes et al. (2002) provides insight to how drug-related harms, like overdose, are not solely attributable to individual behaviors, but rather upheld and perpetuated by an individual’s surroundings [12]. Moreover, PWID-LH may possess additional risks for experiencing an overdose compared to individuals without HIV [14]. Interventions that address surrounding risk environments, rather than focusing on individuals-level risks, have the potential to reduce drug-related harms, as do interventions that address stigma toward people who use drugs [15–18]. However, the Kenya-specific overdose risk environment for PWID-LH has been largely overlooked.”*

4. Pg 6: Can you provide the reasons for declining to participate if known?

We have added this detail in our Methods Section, which showcases the high mobility of PWID populations. The two participants that declined to participate in our study, included on individual that relocated to the coast and another that was taking care of family matters in a rural village, and conveyed they were unsure when they would return to Nairobi. We have modified our statement to the following (Page 5, Lines 161-163):

*“In total, 28 participants were enrolled, with two declining to participate due to permanent and semi-permanent relocations, and four who were unable to be contacted.”*

5. Pg 7: “OAT patients” If this abbreviation is not clarified before please provide its full meaning within the article

We thank the reviewer for their insight and have clarified the meaning of OAT in the Introduction Section, Page 3, Lines 100-104.

“*Harm reduction methods, such as opioid agonist treatments (OAT) and naloxone access, have been foundational in the global response to rising overdose risk [8] and are central to Kenya’s public health agenda. In 2013, Kenya was one of the first countries in Africa to institute a national needle and syringe program to reduce HIV transmission among PWID, and introduced OAT, which is largely methadone, starting in 2014 [9,10].*”

6. It would be beneficial to include the interview script in the paper for the audience's reference. Additionally, could you summarise the main themes and sub-themes in a figure to help readers easily identify them?

The editor partially agreed with this recommendation stating, *“Please note from reviewer 2 comments, from my perspective as editor, the inclusion of the interview script is not required for acceptance but a summary figure with the main themes as sub-themes would be helpful.”* As such, we opted not to include the interview guide, but would be happy to share materials upon request. We have included a summary table of the main themes and brief definitions to accompany each theme, which increases the visibility and transparency of our findings to the PLOS readership. Please note the inclusion of an additional table prompted us to re-number our tables. Please see Table 1, located on Page 8, Lines 221-237.

**Table 1: Overdose (OD) themes and brief definitions**

| ***Themes*** | ***Brief Definition*** |
| --- | --- |
| Experiences of OD | Personal and/or observed experiences following the ingestion or application of a drug or substance that leads to a toxic state or death. |
| OD Precursors | Events directly preceding an OD including taking too much of one substance, injecting unknown substances, polysubstance use, injecting following a period of abstinence, and rushed injections. |
| Safety Strategies | Methods used to prevent an OD, which included avoiding injecting alone, testing batches of drugs in group settings, using small amounts of drugs incrementally, and avoiding polysubstance use. |
| OD Treatment Strategies | Methods applied or witnessed following an OD including first aid techniques aiming to arouse, increase blood flow and oxygenation, and prevent aspiration. |
| Naloxone | Discussions related to the knowledge of, access to and use of Naloxone or other OD reversal medications. |
| Harmful strategies | Non-evidence-based strategies to reverse an OD including a) ingesting cleaning products to expel drugs via vomiting, b) submerging hands and feet in cold water to awaken a person, and c) injecting additional drugs, in an attempt to reverse an OD. |
| Barriers to Treatment | Described as social, political and economic factors such as a) police discrimination, b) medical stigma, c) fatalistic and/or religious views, d) medical and/or transport costs, e) limited access to care, and f) timeliness of OD treatment services. |
| Facilitators to Treatment | Though inconsistently applied, law enforcement agents providing options for drug treatment enrollment, rather than arrest. |

7. If the interview scripts were translated into English, it would be important to acknowledge this as a potential limitation.

Thank you for this suggestion, as we agree that concepts and definitions are easily translated from Swahili to English. We have acknowledged this in our limitations section as follows (Page 24, Lines 643-646):

*“While local research staff with extensive experience working with PWID-LH conducted interviews, transcribed and translated interviews, we acknowledge that translated terms and concepts may not exist in the English language, as they do in Swahili.”*
